# Improving Cattle Health and Welfare in the Area Affected by the First Outbreak of Lumpy Skin Disease in Indonesia

**DOI:** 10.3390/vetsci12090823

**Published:** 2025-08-27

**Authors:** Widi Nugroho, Hani Muhamad Mardani, Ando Fahda Aulia, Achmad Efendi, Michael Philipp Reichel

**Affiliations:** 1 Laboratory of Veterinary Public Health, Faculty of Veterinary Medicine, Universitas Brawijaya, Malang 65145, Indonesia; 2 Department of Animal Husbandry and Veterinary Service, Bureau of Food Security, Bengkalis Regional Government, Bengkalis 28724, Indonesia; mardanidvm@gmail.com; 3 Faculty of Economics and Business, Universitas Riau, Pekanbaru 28293, Indonesia; ando.aulia@lecturer.unri.ac.id; 4 Department of Statistics, Faculty of Mathematics and Natural Sciences, Universitas Brawijaya, Malang 65145, Indonesia; a_efendi@ub.ac.id; 5 Department of Population Medicine and Diagnostic Sciences, Cornell University College of Veterinary Medicine, Ithaca, NY 14853, USA

**Keywords:** cattle shelter, sustainable livelihood framework (SLF), manure disposal, vaccination, serologic survey

## Abstract

This study aimed to investigate cattle farmer livelihoods that relate to cattle welfare in the region with the newly emerging Lumpy Skin Disease (LSD) in Indonesia. A semi-structured interview survey was conducted with randomly selected cattle farmers (n = 102), in Riau. Cattle were bled for analysis of LSD-post-vaccinal seroconversion. The Sustainable Livelihood Framework (SLF) was used; data on livelihood assets, activities, and outcomes were analysed using Multiple Correspondence Analysis (MCA), two-step clustering, and the radar chart of asset possessions. The survey showed that vaccination and veterinary services covered 82.4% and 90.2% of farms. Seroconversion was detectable in vaccinated (15.0%, n = 173) and in non-vaccinated animals (23.1%, n = 13). Farmers mostly fed only grass to cattle (92.2%), with neither pastoral management nor ad libitum water provision. The MCA and cluster analyses indicated that cattle shelter roofing and flooring and manure disposal were the most important markers of the community’s livelihood. Poverty among cattle farmers was 23.5%. The cluster with lower income per capita had lower quality of shelter roofing and flooring, a lack of regular manure disposal, jobless second children, and the lowest possession of natural and physical assets. Helping to possess natural and physical assets might improve cattle farmers’ well-being and cattle welfare.

## Simple Summary

This study aimed to investigate farmer livelihoods associated with cattle welfare in a region affected by newly emerging Lumpy Skin Disease (LSD) in Indonesia. An interview survey was conducted with 102 randomly selected cattle farmers in Siak Kecil, Riau, on topics of livelihood assets, activities, and outcomes. Cattle were bled for analysis of LSD-postvaccinal seroconversion. The survey showed that both vaccination and veterinary services covered the vast majority of farms, but less than one-fourth of vaccinated animals were seroconverted. Yet, non-vaccinated animals were also seroconverted. The vast majority of farmers did not provide ad-libitum water. Cattle shelter roofing and flooring, and manure disposal were the most important markers of the community’s livelihood. Poverty among cattle farmers was almost one-fourth of the population, farmers with lower income per capita had lower quality of shelter roofing and flooring, lacked regular manure disposal, and had the lowest level of possession of natural and physical assets. The thread of LSD remained despite vaccination. Improving cattle shelter quality, managing manure disposal, and helping farmers to possess natural and physical assets might improve farmers’ well-being and cattle welfare.

## 1. Introduction

Lumpy Skin Disease (LSD) is a significant disease of cattle caused by a Capripox virus, which may cause economic lost due reduced milk production, lowered weight gain, condemnation of hide, reduced traction power, reduced manure production, cost of medicine, and extra labour, but it can also cause welfare issues due to morbidity, mortality, painful ulcers across the body surface, and transient infertility [1,2,3,4]. Vaccination is the main control method widely implemented in affected populations [5]. The outbreak of Lumpy Skin Disease of cattle first emerged in Indonesia in 2022, in the Bengkalis region, Riau Province, causing 10.4% morbidity and 0.6% mortality on affected cattle farms [6]. In response to the epidemic, the central government provided a subsidised emergency vaccination program for affected regions, including Bengkalis, using a live attenuated LSDV vaccine, MEVAC™ LSD (MEVAC, Salhya El Gdeda, Egypt) [6].

In Bengkalis, cattle farming was practised by less than five per cent of all households, and the cattle population comprised less than 17 thousand head of cattle, all of beef cattle [7]. The small proportion of cattle farmers in the total community may risk overlooking the health and welfare of cattle by the government, particularly if it comes to priority programs for regional development with a limited budget. The World Organisation of Animal Health used animal-based outcomes as indicators to assess the welfare of the beef cattle production system and proposed a set of recommendations in cattle husbandry practices to optimise the welfare of the animals. These recommendations included resource provisions and activities related to biosecurity and health management (including vaccination), housing, and environment, as well as daily husbandry and handling [8].

Animal health and welfare, however, are often viewed as secondary to the farmer’s well-being, so that livestock welfare is considered when it may improve cattle farming profitability or other means of livelihood security, such as social status [9,10]. In this situation, an animal welfare strategy may need to be placed in the broader context of the farmers’ well-being, and activities may need to be found where the two interests (animal welfare and human well-being) agree with each other, i.e., potential indicators that are considered important for farmers’ well-being but might also contribute to the enhancement of animal welfare.

In studying rural well-being, the most popular method used globally thus far has been the Sustainable Livelihood Framework (SLF) developed by the DFID (Department for International Development) [11,12]. The original SLF consisted of six elements, i.e., vulnerability context; the pentagon of livelihood assets, including social, financial, physical, natural, and human assets; influence and access; transforming structures and processes; livelihood activities (strategies); and livelihood outcomes [13,14]. Critiques of and modifications to the original concept of the framework have been proposed [11,15]. Despite this, many recently published studies still utilise the original SLF to analyse the livelihood of livestock farmers in rural communities worldwide [16,17,18].

On the other hand, a serological survey is a typical activity used to assess the response to vaccination and to update the situation of an animal disease in a region. The information gathered from a survey may give direction on where the resources should be invested to improve animal health outcomes in a region. While the SLF may approach the well-being and welfare from the farmers’ standpoint, the serological survey could assess the performance of veterinary service delivery in the region in improving animal health and welfare outcomes.

The present study used the SLF and a serologic survey, aimed at identifying important indirect indices of cattle welfare that were associated with cattle farmers’ livelihood security. This study was conducted in Siak Kecil, a district with the largest cattle population in the Bengkalis region and affected by the first outbreak of LSD in Indonesia. The knowledge imparted by this study may provide a reference for developing strategies to improve cattle health and welfare, as well as to leverage the well-being of cattle farmers in the region.

## 2. Materials and Methods

### 2.1. Data Collection

#### 2.1.1. Sampling of Farms

This study was conducted in the Siak Kecil District (1.250° N, 102.137° E), which had the largest cattle population among districts in the Bengkalis region. LSD broke out in this district during the period of 24 March–14 May 2022 [6]. The local government recorded, in 2021, a population of 1087 smallholder cattle farms and 3479 individual cattle in the district, all of meat-producing cattle breeds. From this sampling frame, 102 farms were selected randomly, using a random number generation software in Microsoft Excel Windows 11, for a survey from 1 August to 7 October 2022. The sample size gives a 9.3% error at the 95% confidence level for an anticipated frequency of 50%. During the visits, some cattle farmers were not present, not willing to participate, or no longer kept cattle, and in such a case, they were replaced by non-randomly selected cattle farmers living nearest the excluded farmers [10].

#### 2.1.2. The Questionnaire

Data were collected by observation and interviews using semi-structured questionnaires encompassing livelihood assets, livelihood activities, and livelihood outcomes. The actual questionnaire can be accessed in the PDF file format of the Supplementary Materials S1. In total, 25 livelihood assets were included in the interview and observation. Physical capital included seven variables: the area of the house lot; the value of vehicles; the ownership and the financial value of non-cattle livestock and poultry; the number and the financial value of cattle; cattle shelter roofing; cattle shelter partition; and cattle shelter flooring. Human capital included eight variables: age of the farmer; education of the farmer; age of a housewife; education of the housewife; size of a household; number of household members earning money; cattle farm manpower; and cattle farming experience of the farmer. Natural capital included four variables: source of drinking water; the possession of agricultural land; the possession of oil palm land; and cattle feed sources. Social capital included four variables: distance from house to wet market; distance from house to hospital; distance from house to veterinary post; and whether a farmer received LSD vaccination. Financial capital included two variables: possession of insurance and possession of a cash loan.

Livelihood activities involved in the interview and observation included ten variables: the main job occupied by a farmer, housewife, first child, second child, and third child; type of cattle production; means of daily cattle husbandry; means of cattle manure disposal; the use of veterinary services; cattle sale frequency in a year.

The livelihood outcomes asked in the interview included four topics: monthly income of each household member who works to earn money; the total income from cattle sales in a year; the presence of cattle diseases in a year; and the incidence of LSD on the farm during the year. In the Sustainable Livelihood scenario, a disaster, including a disease epidemic, is typically seen as a vulnerability context that leads an affected community to respond by taking actions to collect a set of assets and income to survive [14]. However, cattle diseases, including LSD, could be an outcome of activities and the possession of certain livelihood assets [19]. In this study, data on LSD and other cattle diseases were treated as livelihood outcomes of already existing assets and activities.

#### 2.1.3. Monitoring Antibody Seroconversion Against LSDV

During the visit, along with the interview, blood samples were collected from cattle on the selected farms to monitor the level of antibodies against LSDV in sera. One or two individual cattle were randomly selected in each sample farm and bled, resulting in a total of 186 serum samples from 102 farms, including 173 vaccinated individuals and 13 non-vaccinated animals. Samples were immediately transported on ice to the Laboratory of Veterinary Public Health, Faculty of Veterinary Medicine, Universitas Brawijaya, and kept frozen at −20 °C until the assay was performed. Sera were tested using a competitive ELISA, the ID Screen^®^ Capripox Double Antigen Multi-species ELISA (CPVDA ver 0117 EN, lot K65, IDVet, Montpellier, France), following the manufacturer’s instructions. The >30% S/P cut value was used to deem positive results. Standard negative and positive samples provided by the manufacturer were used as the controls. The ELISA test was conducted at the Central Laboratory of Life Sciences, Universitas Brawijaya, Malang, Indonesia. Data on seroconversion against LSDV were presented descriptively in percentages.

### 2.2. Data Analysis

#### 2.2.1. Conversion of Interview Data

The physical capitals, including the value of vehicles, the value of non-cattle livestock and poultry, and the value of cattle, were converted into the financial values per capita in the household, by dividing the value by the size of the corresponding household. From the data of monthly income of each household member who works to earn money and the total income from cattle sales in a year, a livelihood outcome variable, namely, household income in a year, was estimated by summing the data of the monthly income of all household members, multiplied by 12 months, and adding the data of income from cattle sales in a year. Subsequently, a new livelihood outcome, namely, income per capita per day, was calculated by dividing the total household income in a year by the total number of household members over 365 days. In the further analysis, these two latter livelihood outcome variables were used, instead of the monthly income of each household member who works to earn money and the total income from cattle sales in a year.

Those physical capital values, along with household income in a year and income per capita per day, were converted into USD and aggregated into categories. The average exchange rate during the period of the survey, at USD 1 = IDR 14,952.51, was used. The exchange rate data were obtained from JISDOR (Jakarta Interbank Spot Dollar Rate), accessed on 28 April 2023 from the official website of Bank Indonesia [20] (the update can be accessed at https://www.bi.go.id/en/statistik/informasi-kurs/jisdor/default.aspx). The global absolute minimum poverty line was set by the World Bank in 2011 at USD 1.25 a day [21] and was used to estimate the proportion of households living under the poverty line, based on their income per capita per day. This estimate was used in this study because the value was closer to the locally updated poverty line formally applied by the regional government [22].

Using the data of household members’ main jobs, a new variable in livelihood activity was created, namely, household income source. In this variable, a household was grouped into either of two categories: agricultural or mixed-income household. A household was agricultural if all household members worked in agricultural-related jobs or a mixed-income household if at least one household member worked in a sector unrelated to agriculture.

After the conversions and new variables were created, new sets of 25 livelihood assets, 11 livelihood activities, and 4 outcomes were obtained and used for statistical analyses. All continuous variables in livelihood assets and outcomes were converted into categorical data (Summary-asset-activity-outcome sheet of the xlsx file format of the Supplementary Materials S2).

#### 2.2.2. The Multiple Correspondence Analysis (MCA)

An MCA was conducted to identify important variables among the assets and activities that characterise the cattle farmers’ livelihood. For this purpose, initially, Pearson’s bivariate correlation was performed across assets and activities, to assess the presence of collinearity between the variables. A correlation of >0.6 was used as the threshold for extreme collinearity, and variables with such a characteristic were excluded from further analysis. Subsequently, an MCA was conducted, where assets and activities were used as active variables, while livelihood outcomes were used as supplementary variables. An active variable was deemed important when it had an eigenvalue of ≥0.2 in either of the first two dimensions [23,24].

#### 2.2.3. The Cluster Analysis

A further analysis of two-step clustering was conducted to classify farmers based on important livelihood assets and activities obtained from the first two dimensions of the MCA. Outcomes were used as the evaluation variables in this clustering analysis. The determination of cluster number was performed step-wisely from two to five cluster numbers, and a Silhouette Score was used to determine the best cluster. Furthermore, a Chi-square statistic was used to determine a significant difference between the proportions of categories in different clusters, at a level of 0.05. The largest proportion of a category in a variable was used to name the characteristic of a cluster regarding the variable. Cluster members were visualised in a scatter plot diagram using the MCA object scores in Dimension 1 as the X-axis and in Dimension 2 as the Y-axis. Statistical tests included MCA and the two-step clustering analysis, and they were conducted in the SPSS software (IBM SPSS Statistics for Windows, version 26.0, IBM Corp., Armonk, NY, USA).

#### 2.2.4. The Radar Charts

A radar chart is a popular method used to visualise the five livelihood assets in the SLF. It can display all five assets simultaneously on a single plot, thus facilitating an easy interpretation of how strong or weak each asset is relative to the others. Furthermore, different polygons can be overlaid in one chart, enabling easy comparison among assets in different groups immediately. Radar charts were constructed to depict the magnitude of the possession of assets by cattle farmers in the study area. To this effect, firstly, the weighting of each category in a variable was employed either by referring to previous studies on the corresponding topic or by arbitrary judgment.

In all variables, categories with the highest value were weighted 1 (one). The weight of variables of age assumed that a person between the ages of 30–40 years old was most productive for physical activities, and the productivity score of ages before or after the range would likely to decline by 10% every 10 years [25]. The weight of the size of a household assumed that a larger-sized household benefited from an increased labour force; thus, a larger-sized household was given a higher score [26]. In weighting the cattle shelter flooring, the score of the category “Bare Earth Floor” was deemed zero, the same as the weight of a cattle farm in the category of no shelter provision. It was argued that, in terms of the shelter floor, bare earth had no additional value than if cattle had no shelter. The weight of each category was then multiplied by the proportional frequency of each category to obtain a score for the category. Subsequently, the scores of all categories in a variable were summed to obtain the score of a variable. This variable score and the weight of a variable were used to obtain the final proportional possession of the variable relative to other variables in the same asset group of the SLF. The eigenvalue of a variable was used to weight a variable relative to other variables in the same asset group. Technically, the average eigenvalue of a variable in the first two dimensions was divided by the sum of the average eigenvalues of all variables in the asset group, and multiplied by the variable score, to obtain the proportional possession of the variable in the particular asset group, as shown in Formula (1) below:

(1) $$Y=\frac{\mu i}{\sum \mu }\times Xi$$

where Y = percent of possession of a variable in an asset group; μ*i* = mean of eigenvalue of the first two dimensions of a variable; ∑μ = total of mean of eigenvalue of all variables in the asset group; and X*i* = variable score.

Lastly, proportional possessions of all variables in an asset group were summed to obtain the percentage of the possession of an asset group of the SLF asset pentagon. A radar chart was constructed to visualise the asset pentagon depicting the magnitude of the possession of livelihood asset groups in each of the clusters of cattle farmers obtained from the two-step clustering analysis. 

## 3. Results

### 3.1. Description of Cattle Farming, Vaccination, and Cattle Farmers’ Livelihood Outcomes

During the study with the 102 respondents, there were no missing data identified in any of the variables studied. Data on livelihood assets related to cattle farming showed that, mostly, households had one or two people involved in cattle farming (41.2% or 39.2%), while fewer households had three or four persons involved in cattle farming activities (15.7% or 3.9%). Almost half of the households had >5–10 years of experience in cattle farming (49.0%). One-third of the households had 0–5 years of experience in cattle farming (35.3%), and the least proportion had >10 years in cattle farming (15.7%). The majority of the farmers had <6 cattle per household (77.5%), 20.6% had 6–≤10 heads per household, and only 2.0% of the farmers had >10 cattle per household. Cattle shelter roofing was made of tin in 81.4% of households and thatch in 10.8% of households. Shelter flooring was made of concrete/wood in 49.0% households and bare earth in 43.1% households. The use of cattle bedding was not detected during the study.

Most farmers fed cattle only with grass (92.2%). In addition to grass feeding, a much smaller proportion of farmers also used rice bran, palm kernel meal, or legumes as a feed source for cattle (7.8%). No pastoral grass management was detectable. Instead, the farmers used naturally grown vegetation as cattle feed sources. None of the farmers provided ad libitum drinking water for cattle. Along with cattle farming, keeping small ruminants and poultry was common (78.4% of farmers). The majority of farmers lived ≥10 km from a veterinary post (72.5%). Only 5.9% of farmers lived 5–<10 km away from a veterinary post, and just above one-fifth of farmers lived <5 km from a veterinary post. Vaccination against LSD was delivered to 90.2% of farms.

Data on livelihood activities related to cattle farming were presented. Most households kept cattle for breeding (90.2%). A semi-intensive method of daily husbandry was practised by 80.4% of households, where cattle were let to roam during the day and kept in shelter during the night. Cattle were kept in the shelter all day on 11.8% farms. Eight households did not provide shelter for cattle (7.8%). The farmers commonly collected cattle manure for fertilising oil palm land (75.5%). Cattle were traded at least once by one-third of farmers during a year (36.3%). A fraction of 4,9% of farmers sold cattle twice a year. Despite the majority of the farmers living far from the veterinary post, the active use of regular veterinary services was very common, including disease control and artificial insemination services (82.4%).

Four livelihood outcomes were included in the study. One-sixth of farmers experienced cattle diseases on their farms in a year (17.6%). During the first epidemic season, only 5.9% farmers experienced LSD on their farms, but none of the respondents experienced cattle mortality from LSD or had sold cattle affected by LSD. The survey showed that 33.3% of households had an income per capita of ≥2.50 USD, 43.1% had an income per capita of 1.25–<2.50 USD, and 23.5% had an income per capita lower than the absolute poverty line of 1.25 USD. Cattle sales contributed 15.9% of the total monthly income of the community. If income from cattle sales was omitted from the calculation, the proportion of households living under the poverty line would increase to 28.4%. This difference, however, was not statistically significant (*p* = 0.46). A fraction of 35.3% of households had a household income of <2000 USD per year, 39.2% had a household income of 2000–<4000 USD per year, and 25.5% had a household income of >4000 USD per year. A summary of livelihood assets, activities, and outcomes, including those related to cattle farming, used in the statistical analyses, can be accessed in the Summary-asset-activity-outcome sheet of the xlsx file format of the Supplementary Materials S2.

**Figure 1:**
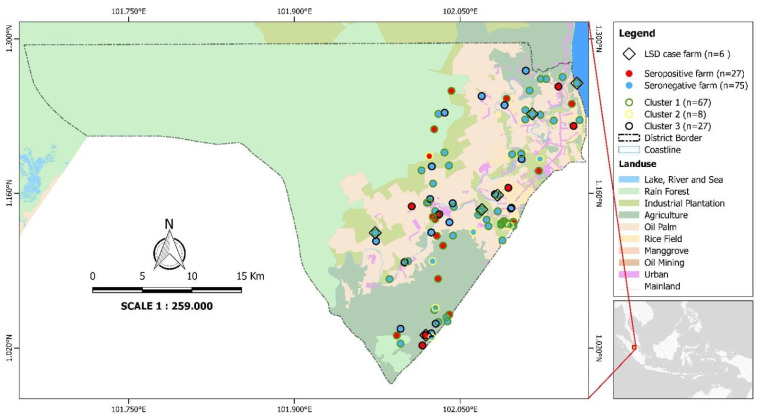
The study area for the livelihood of cattle farmers in a region affected by the first outbreak of Lumpy Skin Disease in Indonesia, 2022. The diamond shape and colours of the border and fill of circles indicate the geographical coordinates of LSD cases, seroconverted farms, and clusters of sampled cattle farming households across the district of Siak Kecil, Riau Province.

The ELISA showed that, among the vaccinated cattle, only 15.0% of animals showed antibody seroconversion (n = 173), and among unvaccinated animals, seroconversion was 23.1% (n = 13). The distribution of seroconversions among sample farms is depicted in Figure 1.

### 3.2. The Multiple Correspondence Analysis

During correlation analysis, three livelihood asset variables of education of housewife, distance from house to hospital, and cattle shelter partition were extremely correlated with more than one variable and, therefore, excluded from the analysis. One livelihood activity variable of means of daily cattle husbandry and the livelihood asset of cattle shelter roofing were detected to be extremely collinear with each other. Because the type of roofing was easier to observe, it was chosen as a variable rather than the type of daily husbandry, for the MCA. After excluding the 4 variables, 22 asset variables and 10 activity variables were included in the MCA analysis as active variables, and the 4 outcomes were treated as supplementary variables, making a total of 36 livelihood variables included in the MCA analysis (Table 1).

The first two dimensions of the MCA explained 29.4% of the total variance of the data. The eigenvalue of active variables showed that six assets and six activities were deemed important in the first two dimensions of the MCA and used in further analyses. They included three human assets: Age of farmer, Age of housewife, Size of household; three physical assets: vehicle per capita (USD), cattle shelter roofing, cattle shelter flooring, and six activities of cattle manure disposal, Housewife’s job, First child’s job, Second child’s job, Third child’s job, and household income source (Table 1). Data on assets, activities, and outcomes of livelihoods used in the MCA analysis are accessible in Supplementary Materials S3.

### 3.3. The Clustering

**Figure 2:**
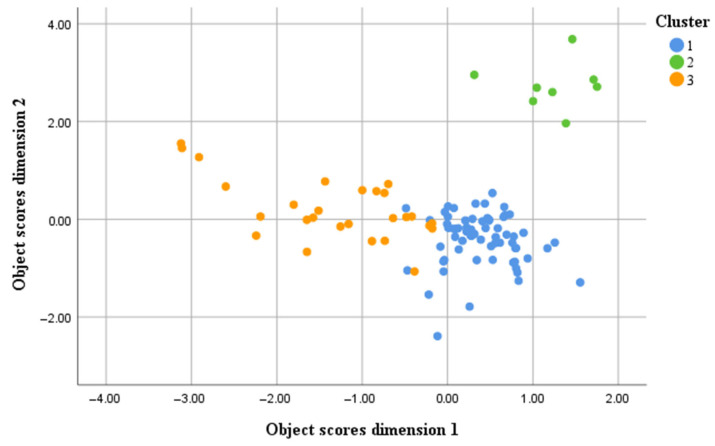
Livelihood clusters of cattle farmers’ community in Siak Kecil, Riau Province, Indonesia, 2022. Cluster 1 was characterised with the use of tin for cattle shelter roofings, the use of wood/concrete for cattle shelter floorings, the use of cattle manure for fertiliser, having household with a size of 4–<7 persons, having three children who are all jobless, and having a farmer and housewife at ages of 40–50 years old. Cluster 2 had characteristics of having no cattle shelter and a lack of the collection of cattle manure for fertiliser. This cluster was similar to Cluster 1 in terms of having a household with a size of 4–<7 persons, having three children who are all jobless, and having a farmer and a housewife at the ages of 40–50 years old. Cluster 3 was farmers with a cattle shelter roof made of tin, a cattle shelter floor from wood/concrete, and the use of cattle manure as fertiliser. Households in this cluster mainly live with no child with them, with a household size of <4 persons, and with a farmer of ≥60 years old and a housewife of 50–<60 years old.

A three-cluster solution was deemed a good clustering number, based on the largest Silhouette Score at 0.3, indicating a fair clustering quality. Figure 2 depicts that the clustering, fairly demarcated Clusters 1 and 3, while Cluster 2 was well discriminated from the other two. Table 2 indicates that four variables were able to predict the clusters at the prediction score of ≥0.79, namely, variables of Cattle shelter roofing, Cattle shelter flooring, Cattle manure disposal, and Second child’s job. The other variables had scores of predictive importance of less than 50%.

Cluster 1 was characterized by the use of tin for cattle shelter roofings, the use of wood/concrete for cattle shelter floorings, the use of cattle manure for fertiliser, having household size of 4–<7 persons, having three children who are all jobless, and having farmer and housewife at ages of 40–50 years old. Farmers in this cluster had an income per capita of 1.25–<2.50 USD and had an annual household income of 2000–<4000 USD.

Cluster 2 had characteristics of having no cattle shelter and a lack of a collection of cattle manure for fertiliser. This cluster was similar to Cluster 1 in terms of having a household size of 4–<7 persons, having three children who are all jobless, and having a farmer and a housewife at the ages of 40–50 years old. However, households in this cluster had an income per capita of <1.25 USD and had an annual household income of <2000 USD.

Cluster 3 was farmers with cattle shelter roofing made of tin, cattle shelter flooring from wood/concrete, and the use of cattle manure for fertiliser. Households in this cluster mainly lived with no child with them, with a size of household < 4 persons, and with a farmer of ≥60 years old and a housewife of 50–<60 years old. They had an income per capita of 1.25–<2.50 USD and had an annual household income of <2000 USD.

### 3.4. The Radar Charts Visualisation

**Figure 3:**
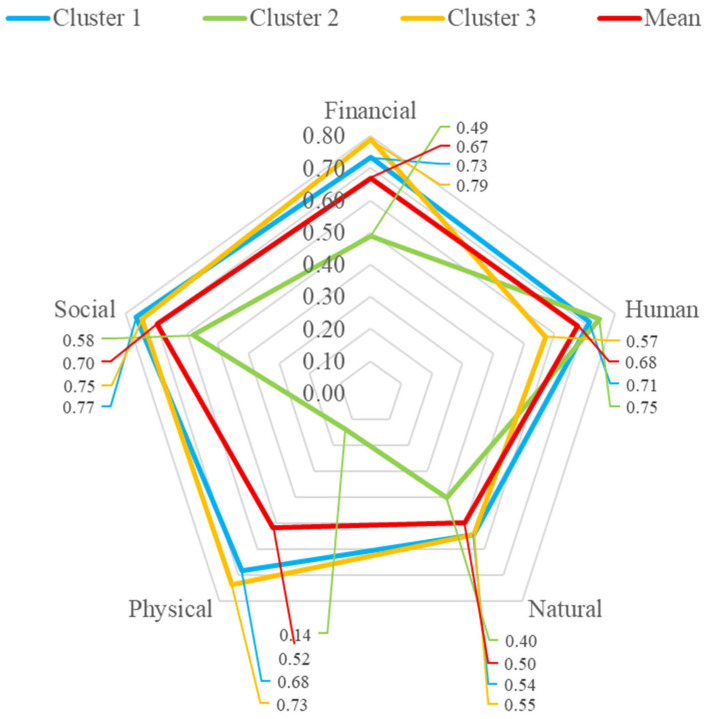
Radar chart of the livelihood asset pentagon of cattle farmers in Siak Kecil, Riau, Indonesia, 2022. Cluster 2 has a marker characteristic of the lowest fraction of physical, natural, financial, and social assets among the other clusters.

The radar chart of variable scores of assets is shown in Figure 3. Among the five asset groups, the possession of social assets was the highest, followed by human, financial, physical, and natural assets (70%, 68%, 67%, 52%, and 50%). The radar chart shows that Cluster 1 had the largest proportion of social assets (77%), and the proportion of other assets was in between the other two clusters. Cluster 2 had the highest proportion of human assets among the clusters (75%) but the lowest proportion in all four other asset groups, especially the physical asset, in which this cluster had a very low proportion (14%). Conversely, Cluster 3 had the lowest in the possession of human assets (57%) but the largest proportion of possession in financial, social, physical, and natural assets among other clusters (77%, 75%, 73%, and 55%). Detailed calculation of the percent of possession of a variable in an asset group is available in the Radar chart sheet of the xlsx file format of the Supplementary Materials S2.

## 4. Discussion

This study indicated that the clinical incidence of LSD was low at 5.9% of the farms at the district level, and the mortality was undetectable during the survey. This confirmed the previous outbreak investigation study at the regional level in Bengkalis that reported that 1.2% of farms were affected, and mortality occurred on less than 0.14% of total farms [6]. Forced sales of diseased animals were undetected; this might be due to low morbidity and fatality during the outbreak. Despite long distances between the majority of farms and the veterinary post, both the utilisation of veterinary services and the coverage of vaccination were high, indicating the functionality of the services as a means of resource provision to maintain animal health and welfare in the study area.

The proportional LSDV seroconversion, however, was small after three months in vaccinated individuals. This was in accordance with a study from a field trial in Vietnam, which used the same vaccine product and detection kit as in the current study, which reported a low proportion of seroconversion after five months post-vaccination [27]. The small proportion and short-lived seroconversion in the current study were speculated to be due to the low vaccine quality. However, the Vietnamese observational study claimed a protection level of more than 80% within a year, and the immunity was cell-mediated. On the other hand, other studies showed that there seemed to be seasonality for the LSD outbreak [6,28]; thus, the lack of incidence in the second semester of the year after vaccination could also be due to this seasonality. A challenge study is needed in animals, five months following vaccination, to confirm the hypothesis of cell-mediated immunity.

Furthermore, unvaccinated cattle also showed seroconversions, indicating subclinical infections and that the wild virus remained circulating in the environment, posing a threat of the re-emergence of the outbreak. For practical reasons, annual vaccination, advisably at the beginning of the anticipated risk period of the rainy season, may be required to prevent the subsequent outbreak of LSD.

The vast majority of farmers provided shelters for cattle, but nearly half used bare earth for flooring. Furthermore, wealthier farmers tended to build their cattle shelters with better quality, with concrete flooring and tin roofing. They also tended to manage cattle manure better by regularly disposing of it for fertilising oil palm lands, which is a typical practice to reduce fertiliser costs in the area [29,30]. A good shelter protects cattle from the cold or the heat stress, and ideal shelter flooring provides a clean, sanitary, warm, comfortable, and dry environment [8]. Moreover, cattle manure disposal may prevent the accumulation of pathogens, such as Foot and Mouth Disease virus and *Salmonella* sp. [31,32], and may prevent the breeding of stable flies [33], which is among the competent biological vectors of LSD [34]. As the cattle shelter and manure disposal were important characteristics of cattle farmers, encouraging farmers to improve shelter quality and manure disposal could be among the starting points that improve farm biosecurity, leading to improved cattle health and welfare. In turn, it could help to enhance the productivity of cattle, as well as farmers’ well-being.

The other important husbandry practices, which were challenging to implement in the study area, were the ad libitum provision of drinking water for cattle and the improved quality of nutrition. Although the majority of farmers had long cattle farming experience of more than five years, knowledge of quality food remained in question, as no quality pastoral management was detectable, and only less than ten per cent of farmers used supplementary feeds in addition to grass feeding. In our experience working with local farmers, there was a belief that excessive drinking could cause cattle diarrhea. While it could be true when water was contaminated with pathogens or toxins [35], the fear of acute diarrhea has been distracting farmers from the less visible infertility consequences of dehydration [1]. Systematic cattle husbandry training, probably conducted in a farm model with good husbandry practices, should be part of the future program to improve cattle health, welfare, and production in the region. Furthermore, the percentage of farms with morbidity was low in this study. This was surprising and raised questions regarding whether subclinical diseases, such as internal parasitism or some mild diarrhea, were prevalent but went unnoticed by farmers. Preweaning morbidity and mortality, stunting, and infertility were not captured in the current study either and should be the subject of future studies on cattle welfare and health in the region.

This study revealed that the fraction of poverty among cattle farmer households was almost four times that of the poverty in the Bengkalis region, which was reported low at 6.32% at a threshold of income per capita of approximately 1.43 USD set up by the local government [22]. This suggests that poverty alleviation programs in the region should be addressing the cattle farmer community as a priority. The MCA and cluster analyses further indicated that the presence of a jobless second child in a household was a significant marker of the deeper poverty level of the household. Helping young household members obtain jobs could be among the focal points for livelihood improvement programs. Alternatively, if they were young households, yet with a couple of children as a result of young marriage [36], more complex assistance would be needed. Provided wealthier farmers tended to look after cattle better by providing good shelter and manure management, poverty alleviation in general could be an indirect pathway to improve cattle welfare related to shelter provision in the region.

Furthermore, with respect to livelihood security, the radar chart of assets indicated that households with the highest income per capita possessed more financial, natural, and physical assets than other clusters. Conversely, households with the lowest level of income had the most limited possession of financial, social, and especially natural and physical assets but the highest fraction of human assets. Human assets alone did not seem enough to leverage the income per capita of these households in the study area. Attempts are needed to help unfortunate households to possess more natural and physical assets as a pathway to lift them from poverty. One strategy might be to nurture young household members to collect physical and natural assets. Possession of natural assets in terms of land could also, in turn, facilitate the provision of quality grass for cattle.

Finally, despite careful study design, results drawn from this study should be used with caution due to some limitations. This study was conducted in one district in Indonesia; thus, the result may not apply to other regions. Data on variables such as income, water provision, and manure management relied on farmer self-reporting were subject to variability in perception and inaccuracy of recall. Non-random selection of replacement farmers might cause bias in sampling selection.

## 5. Conclusions

In conclusion, this study indicated that the resource provisions to improve cattle welfare, i.e., better cattle shelter and manure disposal, were important parts of the daily livelihood of farmers in the study area. Veterinary service and vaccination program as a means of resource provision to maintain cattle welfare were already well accessed by the vast majority of farmers. The threat of the LSD outbreak remained; apart from vaccination, encouraging the practice of regular manure disposal could provide a non-medical method to improve farm biosecurity against LSD and other manure-borne diseases of cattle. Poverty was high among cattle farmers. Helping farmer households with the possession of more natural and physical assets and paid jobs for young household members could become a pathway to leverage the livelihood of farmers in the study area and the welfare of their cattle.

## Figures and Tables

**Table 1:** The first two dimensions of Multiple Correspondence Analysis of 36 components of the livelihood of cattle farmers in Siak Kecil, Riau, Indonesia, 2022. * indicates an important active variable of the dimension at a threshold of >0.2; ^a^ indicates a supplementary variable.

Livelihood Component	Variable	Eigenvalue of Dimension		Mean
		1	2	
Financial	Having insurance	0.056	0.035	0.045
	Having a cash loan	0.000	0.005	0.003
Human	Age of the farmer	0.326 *	0.155	0.240
	Education of the farmer	0.049	0.048	0.049
	Age of the housewife	0.582 *	0.069	0.326
	Size of household	0.401 *	0.026	0.213
	Household members earn money	0.061	0.175	0.118
	Cattle farming manpower	0.104	0.127	0.115
	Cattle farm experience	0.028	0.113	0.071
Natural	Asset oil palm land per capita (Hectare)	0.177	0.163	0.170
	Drinking water source	0.074	0.013	0.043
	Agricultural land ownership	0.000	0.013	0.006
	Cattle feed source	0.040	0.041	0.041
Physical	Asset house lot per capita m^2^	0.109	0.142	0.126
	Asset vehicle per capita USD	0.277 *	0.005	0.141
	Asset non-cattle livestock and poultry per capita USD	0.003	0.049	0.026
	Asset cattle per capita USD	0.182	0.103	0.142
	Cattle shelter roofing	0.220 *	0.667 *	0.444
	Cattle shelter flooring	0.151	0.648 *	0.399
Social	Distance from the house to the wet market	0.059	0.131	0.095
	Distance from the house to the veterinary post	0.009	0.064	0.036
	Received LSD Vaccination	0.002	0.043	0.023
Activity	Type of cattle production	0.018	0.025	0.022
	Cattle manure disposal	0.136	0.655 *	0.396
	Cattle sale frequency	0.026	0.045	0.035
	Use of vet service	0.010	0.009	0.009
	Farmer’s job	0.053	0.154	0.104
	Housewife’s job	0.389 *	0.100	0.245
	First child’s job	0.622 *	0.102	0.362
	Second child’s job	0.626 *	0.096	0.361
	Third child’s job	0.239 *	0.063	0.151
	Income source	0.056	0.235 *	0.146
Outcome	Income per capita per day USD ^a^	0.031	0.070	0.050
	Household income a year USD ^a^	0.024	0.050	0.037
	LSD case ^a^	0.001	0.004	0.002
	Disease case ^a^	0.000	0.005	0.002
% of Variance		15.889	13.496	14.693

**Table 2:** Two-step clustering of cattle farmer livelihood in Siak Kecil, Riau Province, Indonesia, 2022. ^a^ Field evaluation of the clusters; * Significant at 0.01.

Variable	Predictor Importance	Cluster 1	Cluster 2	Cluster 3	*p*-Value
		(n = 67)	(n = 8)	(n = 27)	
Cattle shelter roofing	1	Tin	no shelter	tin	<0.01 *
Cattle shelter flooring	0.95	wood/concrete	no shelter	wood/concrete	<0.01 *
Manure disposal	0.95	used for fertiliser	no collection	used for fertiliser	<0.01 *
Second child’s job	0.79	jobless	jobless	no second child	<0.01 *
Size of household	0.42	4–<7	4–<7	<4	<0.01 *
First child’s job	0.31	jobless	jobless	no first child	<0.01 *
Third child’s job	0.27	jobless	jobless	no third child	<0.01 *
Age of housewife	0.24	40–50	40–50	50–<60	<0.01 *
Age of farmer	0.19	40–50	40–50	≥60	<0.01 *
Income source	0.16	Agricultural	Agricultural	Agricultural	<0.01 *
Housewife’s job	0.04	Homemaker	Homemaker	Homemaker	>0.05
Asset vehicle (USD)	0.04	<455	<455	<455	>0.05
Household income a year (USD) ^a^	0.1	2000–<4000	<2000	<2000	<0.01 *
Income per capita per day (USD) ^a^	0.05	1.25–<2.50	<1.25	1.25–<2.50	>0.05
LSD case ^a^	0.01	No	No	No	>0.05
Disease case ^a^	0.01	No	No	No	>0.05

## Data Availability

The original contributions presented in this study are included in the article/Supplementary Material. Further inquiries can be directed to the corresponding author(s).

## Supplementary Materials

The following supporting information can be downloaded at: https://www.mdpi.com/article/10.3390/vetsci12090823/s1, and the actual questionnaire can be accessed in PDF file format of the Supplementary Materials S1. Summary description of assets, activities, and outcomes used in the statistical analyses, as well as the calculation procedure to obtain variable scores for the radar chart visualisation, can be accessed in the xlsx file format of the Supplementary Materials S2. Data used in the statistical analysis can be accessed in sav file format of the Supplementary Materials S3. The file of the original questionnaire responses can be accessed upon request to the corresponding author.
