# Supplementary material for: Improving Cattle Health and Welfare in the Area Affected by the First Outbreak of Lumpy Skin Disease in Indonesia
Source: Vet Sci. 2025 Aug 27;12(9):823. doi: 10.3390/vetsci12090823 (PMC12474218; doi:10.3390/vetsci12090823)
Supplement: Supplementary file 1 [file vetsci-12-00823-s001.zip › Supplementary Materials S1.pdf]

Questionnaire
Livelihood security and cattle welfare in Bengkalis, 2022

No _____________
Date _____________
Geographic coordinate Lat__________Long___________

livelihood assets
Physical capital
1. The area of the house lot __________________m²
2. The total value of vehicles IDR ________________
3. The ownership and the financial value of non-cattle livestock and poultry
    - Goat and sheep ____________heads, total value IDR ________________
    - Poultry (including chicken, duck, geese) ________ heads, total value IDR_________
4. Cattle ____________heads, total value IDR__________________
5. Cattle shelter roofing (tin/tarp/thatch/ _______________)
6. Cattle shelter partition (wood/concrete/ _______________)
7. Cattle shelter flooring (bare earth/wood/concrete/ _______________)

Human capital
1. age of farmer __________years old
2. education of farmer
   (unschooled/primary/junior high school/senior high school/university)
3. age of housewife __________years old
4. education of housewife
   (unschooled/primary/junior high school/senior high school/university)
5. size of household ____________person
6. number household members earning money ____________person
7. cattle farm manpower ____________person
8. cattle farming experience of the farmer _____________years

Natural capital
1. source of drinking water (rain/ground water well/purchase/________________)
2. possession of agricultural land (yes/no)
3. possession of oil palm land ___________Hectare
4. cattle feed sources (________/________/________/________/__________/__________)

Social capitals
1. distance from house to wet market ______________Km
2. distance from house to hospital ______________Km
3. distance from house to veterinary post ______________Km
4. received LSD vaccination (yes/no)

Financial capital
1. possession of insurance (yes/no)
2. possession of a cash loan (yes/no)

Livelihood activities
1. the main job occupied by the farmer ___________________

2. the main job occupied by the housewife ____________________
3. the main job occupied by the first child ____________________
4. the main job occupied by the second child ____________________
5. the main job occupied by the third child ____________________
6. type of cattle production (breeding/fattening/mix/_________)
7. means of daily cattle husbandry (intensive/semi-intensive/extensive)
8. means of cattle manure disposal (for fertiliser/let sit behind the shelter/______________)
9. the use of veterinary services (yes/no)
10. cattle sale frequency in a year (once/twice/_________)

The livelihood outcomes

1. monthly income of each household member who works to earn money
   - The farmer IDR________________
   - The housewife IDR________________
   - The first child IDR________________
   - The second child IDR________________
   - The third child IDR________________
   - Total IDR________________
2. total income from cattle sales in a year IDR________________
3. experience cattle disease incidence in a year (yes/no)
   - if yes (diarrhea/skin disease/respiration/bloat/lameness/fever/______/______/______)
4. the incidence of LSD on the farm during the year (yes/no)
   - death due to LSD _______________heads
   - forced sales due to SLD _______________heads
